# Comparison of Cartilage Mechanical Properties Measured During Creep and Recovery

**DOI:** 10.1038/s41598-020-58220-2

**Published:** 2020-01-31

**Authors:** Hattie C. Cutcliffe, Louis E. DeFrate

**Affiliations:** 1 Department of Orthopaedic Surgery, Duke University, Durham, NC USA; 2 Department of Biomedical Engineering, Duke University, Durham, NC USA; 3 Department of Mechanical Engineering and Materials Science, Duke University, Durham, NC USA

**Keywords:** Cartilage, Osteoarthritis

## Abstract

The diagnosis of osteoarthritis (OA) currently depends on the presence of pain and radiographic imaging findings, which generally do not present until later stages of the disease when the condition is difficult to treat. Therefore, earlier detection of OA pathology is needed for improved disease management. *Ex vivo* cartilage studies indicate that changes in the mechanical function of cartilage occur as degeneration progresses during OA. Thus, measurement of the *in vivo* cartilage mechanical response may serve as an earlier indicator of OA pathology. Though mechanical characterization is classically performed during loading, the unloading (recovery) response of cartilage may also enable determination of mechanical response. Therefore, the purpose of this study was to validate the use of the recovery response for mechanical characterization of cartilage in a controlled, *ex vivo* environment. To do so, confined compression creep and recovery tests were conducted on cartilage explants (N = 10), and the resulting mechanical properties from both the creep and recovery phases were compared. No statistically significant differences were found in the mechanical properties between the two phases, reinforcing the hypothesis that unloading (recovery) may be a good surrogate for loading.

## Introduction

Osteoarthritis (OA) is a degenerative disease of articular cartilage and affects over 27 million Americans^1^. OA is currently diagnosed via the presence of pain and radiographic features, such as osteophytes and joint space narrowing, from which cartilage loss is inferred^2–4^. Unfortunately, radiographic imaging techniques rely upon gross morphological changes to be present in the tissue, which may not occur until late in the disease^2^. Further, gross morphological changes visible on radiography do not always correlate with pain or functional impairment^3^. As an alternative, magnetic resonance imaging (MRI) has been used to assess OA^5^; however, radiography remains the traditional modality for clinical OA assessment and diagnosis^2,3^. Currently, treatment for end-stage OA is limited and includes pain management or joint replacement surgery to restore function and reduce pain^3^. While these treatment strategies ameliorate symptoms associated with the disease, they do not directly treat or reverse cartilage degeneration. Therefore, earlier detection of OA degeneration is needed for more effective disease management and treatment.

Prior to gross morphological changes, other changes occur in cartilage tissue during the progression of OA^6^. These include compositional changes, such as the loss of proteoglycan content^7–9^, the loss of collagen content and organization^9,10^, and changes in tissue hydration^6,8^. These changes alter the tissue’s response to mechanical load and thus its mechanical function^8,9,11–14^. Altered mechanical function may also contribute to further pathology and degeneration within the tissue^11^. As such, mechanical response may represent a biomarker of OA pathology. Because these compositional and mechanical changes potentially occur prior to the onset of pain or gross imaging findings, detection of these mechanical changes may lead to an earlier diagnosis of OA.

Cartilage mechanical assessment is classically performed in the *ex vivo* environment by excising explants of cartilage tissue, or by exposing the cartilage surface and indenting upon it^15–18^. As such, *in vivo* application of these techniques is limited, especially as diagnostic or prognostic tools. On the other hand, previous work has used MR imaging to measure *in vivo* cartilage response by quantifying changes in cartilage volume and thickness^19–35^. In these studies, an exercise activity is used to mechanically load the cartilage tissue, and MR images from before and after the activity are compared to quantify the resulting deformation. This technique is possible due to cartilage’s viscoelastic nature^36,37^. During loading, cartilage exhibits time-dependent behavior, which is primarily due to water flow out of the tissue^37–39^ but is also due in part to the intrinsic viscoelasticity of the extracellular matrix^40–44^. Because of this time-dependent behavior, MR images can be used to measure load-induced deformation, such as changes in cartilage thickness or volume^19,20^.

Furthermore, due to the viscoelastic nature of cartilage tissue, mechanical characterization of cartilage requires knowledge of the strain-time history. Therefore, cartilage’s mechanical response may be quantified *in vivo* using this MRI methodology by assessing deformation repeatedly after different intensities or doses of exercise. For example, prior work by our group^45^ varied the duration and intensity of walking to quantify how cartilage strain changed over time after walking. This was analogous to a creep test, where the load was applied and held for a set duration, and the resulting strain was measured across time during that duration. However, this paradigm—repeated applications of pre/post MR imaging and exercise—led to an expensive and time-intensive study.

**Figure 1:**
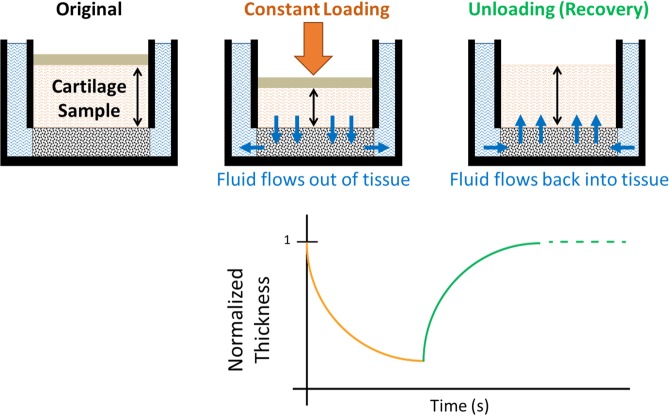
Comparison of loading and unloading (recovery) response of cartilage.

Alternatively, adapting this current *in vivo* MRI methodology^45^ to assess the reversal of cartilage strain during the recovery period after loading may lead to a more efficient study design, while still enabling assessment of the mechanical response (Fig. 1). As previously stated, water flows out of the tissue during loading and deformation accumulates in a time-dependent manner within the cartilage^37–39^. Conversely, upon the removal of load during the recovery period, water flows back into the tissue, causing the reversal of the deformation over time as the cartilage returns to its baseline state. If the unloading trajectory is similar to the loading trajectory, then measuring the strain history during recovery should allow for similar mechanical characterization as measuring the strain history during creep.

Therefore, the purpose of this study was to measure and compare the mechanical response of healthy cartilage to loading (creep) and unloading (recovery). Specifically, the mechanical response was studied in a controlled *ex vivo* environment by conducting confined compression creep and recovery tests on porcine cartilage explants from the tibial plateau and femoral trochlea. Our goal was to compare the strain trajectories during loading and unloading to determine whether the recovery response may be used as a surrogate measure for the creep response when making mechanical assessments of healthy cartilage tissue *in vivo*. This represents a first step toward understanding whether measurements of recovery in OA cartilage may serve as earlier indicators of cartilage pathology.

## Results

**Figure 2:**
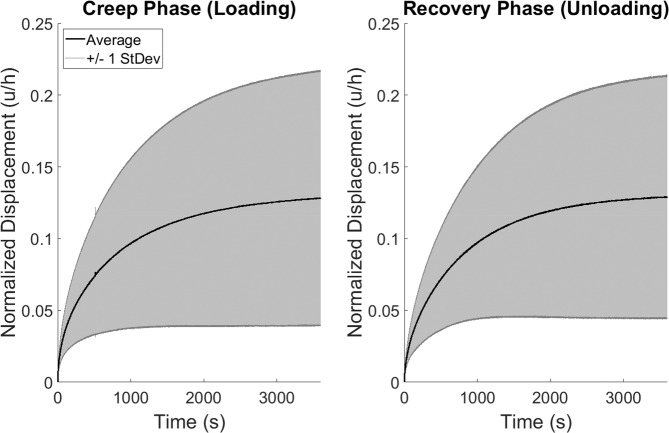
Creep and recovery deformation response corridors (mean ± 1 standard deviation) across all explants (N = 10). Mechanical properties (aggregate modulus and characteristic time) were not statistically significantly different between the creep and recovery phases.

Overall, explants in this experiment experienced a mean strain of 12.8% ± 8.9% at creep equilibrium (end of the creep phase). Explants recovered to 99.0% ± 0.9% of their baseline thickness by the end of the recovery phase (baseline thickness: 0.75 ± 0.18 mm). Furthermore, no statistically significant differences were found in mechanical properties between the creep and recovery phases (Fig. 2). The mean aggregate moduli were 0.71 ± 0.50 and 0.68 ± 0.48 MPa (creep and recovery, respectively), and the mean characteristic times (Eq. 1c, a measure of how quickly equilibrium is reached) were 11.6 ± 5.2 and 12.6 ± 7.3 min, respectively. Cartilage location (tibial plateau vs femoral trochlea) had a statistically significant main effect on both of the mechanical properties (aggregate modulus and characteristic time) measured in this study.

Furthermore, statistically significant correlations were found between the aggregate moduli of each phase (Pearson r = 0.996), and between the characteristic times of each phase (Pearson r = 0.897) (Table 1). Additionally, the characteristic times of each phase were significantly correlated with the aggregate moduli of each phase, respectively (Table 1).

**Table 1:** Pearson Correlation Coefficients Between Outcome Variables.

		Aggregate Modulus		Characteristic Time	
		Creep	Recovery	Creep Time	Recovery
Aggregate Modulus	Creep	r = 1.000	r = 0.996, (p < 0.0001)	r = −0.851, (p < 0.0018)	r = −0.720, (p < 0.0188)
	Recovery		r = 1.000	r = −0.843, (p < 0.0022)	r = −0.718, (p < 0.0194)
Characteristic Time	Creep			r = 1.000	r = 0.897, (p < 0.0004)
	Recovery				r = 1.000

**Figure 3:**
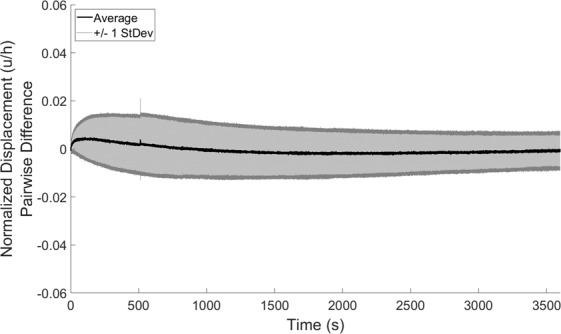
Pairwise differences in deformation response (creep minus recovery) at each point in time (mean ± 1 standard deviation) across all explants (N = 10).

Lastly, the mean average residual between creep and recovery phases in this experiment represented an error of 5.4% ± 3.3% of the final (60 minute) creep strain, indicating a high degree of similarity between the creep and recovery responses in the confined compression environment. Indeed, a calculation of the pairwise differences between creep and recovery at each point in time (Fig. 3) illustrates that the mean pairwise difference remained low at all times.

## Discussion

The mechanical response of cartilage changes as the tissue progresses from a healthy to a degenerated state, such as during OA pathology^6–10,46^. Therefore, measurement of the tissue’s mechanical response may serve as a useful biomarker of cartilage disease. Because traditional methods for the characterization of mechanical response are invasive when applied to cartilage tissue, they are not well suited for *in vivo* applications. Therefore, the development of a method to noninvasively assess the cartilage mechanical response *in vivo* is needed, which may be accomplished using MRI^45^. To this end, the current study evaluated the use of the recovery strain trajectory as a surrogate for the creep strain trajectory in healthy cartilage in a controlled, *ex vivo* environment, in order to validate the assessment of healthy cartilage recovery for quantifying *in vivo* mechanical properties.

Importantly, this study did not find statistically significant differences in either the aggregate modulus or the characteristic time between the creep and recovery phases. This finding motivates the use of the recovery phase as a surrogate for the creep phase, especially for measurement of bulk properties or response such as the total (engineering) strain within the tissue, as measured in the current study. This is useful as classically, mechanical properties are assessed during the loading response, but the unloading (or recovery) response is more accessible *in vivo* using MRI techniques. Therefore, understanding whether the recovery response reflects the loading response *ex vivo* is crucial for validating this *in vivo* approach. Moreover, the mean characteristic times measured in this study, 11.6 ± 5.2 min for creep and 12.6 ± 7.3 min for recovery, are on the same order as those measured *in vivo*. Specifically, in a prior study^45^, the mean *in vivo* creep trajectory of healthy human tibial cartilage had a characteristic time of 17.2 minutes. While future exploration of cartilage recovery *in vivo* is needed to determine whether the *in vivo* creep response may be represented by the *in vivo* recovery response, the findings of the current study support this idea.

Additionally, the current study found that the average residual, defined as the expected error between the creep and recovery response at any point in time, was low: it represented 5.4% ± 3.3% of the final (60 minute) creep strain. However, the average residual was not zero. Further, larger pairwise differences were seen at early time points (0–800 seconds) than at later time points (Fig. 3). These observations are consistent with prior literature, which also did not find the creep and recovery phases to be perfectly reversible^39,47–50^. These differences may be due in part to the extracellular matrix’s intrinsic viscoelasticity under compression^41,44^. However, it may be that these slight differences are due to strain-dependent permeability^51^, in which the tissue’s permeability decreases as the strain in the tissue increases. A decrease in permeability indicates more resistance to fluid flow, making it more difficult for fluid to enter or leave the cartilage matrix. Therefore, at early times in the creep response, there is little strain within the tissue and the permeability is near its maximum, making it easier for fluid to flow out of the tissue and strain to accumulate in response to the sustained load. Conversely, at early times in the recovery response, there is a large amount of strain in the tissue (the strain is near its maximum) and the permeability is lower, making it more difficult for fluid to flow back into the tissue and for strain to dissipate. This is supported by our observations of the pairwise differences (Fig. 3), which we defined as the creep response minus the recovery response at each point in time. The positive values at early times (0–800 seconds) indicate that the creep deformation was typically larger than the recovery deformation at these early times, which is consistent with a larger relative permeability at this point in creep than in recovery. In the current study, the permeability was assumed to be constant (Eq. 1, see Methods section). In the presence of strain-dependent permeability, a slower recovery is expected due to a large amount of tissue compaction and minimal permeability at early times, indicating that longer times are needed to reach equilibrium in the recovery phase. Indeed, this was also observed in the current study, as the characteristic recovery time was typically longer than the characteristic creep time (11.6 ± 5.2 min for creep and 12.6 ± 7.3 min for recovery), though these differences were not significant. Future studies investigating incorporation of a strain-dependent permeability term when modeling the creep and recovery deformation would be beneficial to test this hypothesis. Nonetheless, the observed pairwise differences are small, especially at later times throughout the deformation ( > 800 seconds), leading to a small average residual and nonsignificant differences in the creep and recovery characteristic times.

In this study, statistically significant differences in mechanical properties between cartilage from the tibial plateau and femoral trochlea were found, which is consistent with prior literature indicating that regional differences exist in mechanical properties (for example, between femoral and tibial cartilage)^47,48,52–54^. Porcine tibial and femoral cartilage was found to have aggregate moduli during creep of 1.17 ± 0.48 and 0.40 ± 0.18 MPa, respectively. Likewise, the creep characteristic times were 7.4 ± 3.2 and 14.4 ± 4.4 min, respectively. Further, these mechanical property values—both for the modulus and characteristic time—are consistent with literature values of *ex vivo* cartilage mechanical properties measured via confined compression^9,14,54–56^. Finally, upon reaching recovery equilibrium (end of the recovery phase), explants recovered to within 99.0% ± 0.9% of their baseline thickness, confirming previous reports of thickness recovery upon load removal in *ex vivo* creep experiments^39,47–49^. Future work is needed to expand on these results by testing degenerated or OA cartilage, to understand whether pathology affects the loading response in the same manner as it affects the unloading response.

Overall, measuring the recovery response may be a useful surrogate for the creep response, especially in the *in vivo* context of measuring cartilage mechanical function where the recovery phase is more accessible via MRI than the loading phase. This study investigated recovery of healthy cartilage in the controlled *ex vivo* environment of confined compression, and did not find statistically significant differences in cartilage mechanical properties (aggregate modulus and characteristic time) between the creep and recovery phases. These results help validate the use of the recovery response to measure *in vivo* mechanical properties, with the eventual goal of leveraging mechanical changes as prognostic or diagnostic indicators of cartilage degeneration. The findings of this study motivate the investigation of creep and recovery in degenerated cartilage to further validate whether the recovery response is indicative of the creep response in OA, and whether mechanical changes may represent earlier indicators of cartilage degeneration than pain or radiographic findings.

## Materials and Methods

### Mechanical testing

*Ex vivo* confined compression creep and recovery experiments were carried out using full-thickness cartilage explants (N = 10) from porcine femurs and tibiae. Skeletally mature porcine knee joints were obtained intact from already deceased animals from a local abattoir, so Institutional Animal Care and Use Committee (IACUC) approval was not required. Joints were dissected to expose the articular cartilage surfaces of the femur and tibia, and 5 mm diameter cartilage explants were harvested from the medial and lateral tibial plateaus and medial and lateral femoral trochlea of the joints. Explants were harvested from visually healthy regions of cartilage, identified as areas with a Collins grade of 0^57^. After harvest, explants were promptly wrapped in PBS-soaked gauze and stored at −20 °C until testing.

**Figure 4:**
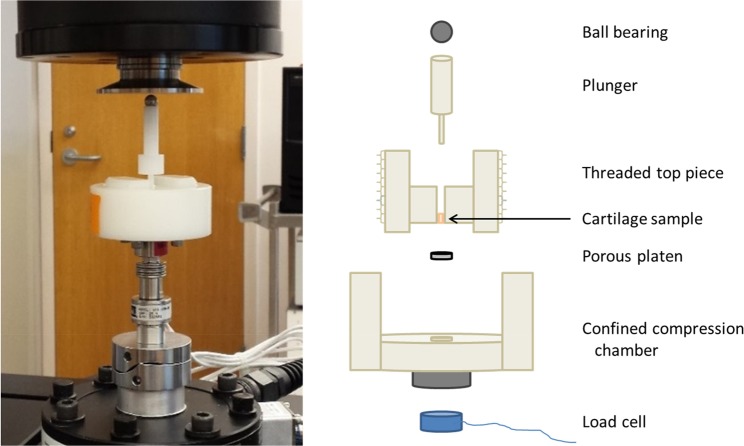
Confined compression fixturing.

Explants were thawed at room temperature for 30 minutes prior to mechanical testing (as a result, explants experienced only one freeze-thaw cycle in this experiment). After thawing, 3 mm diameter cylindrical plugs were cut from the original 5 mm diameter explants and loaded into a confined compression chamber filled with PBS for mechanical testing (Fig. 4). The diameter of the cylindrical chamber within which the explants were loaded was 3 mm, resulting in a very tight fit between the explant and the lateral walls of the confined compression chamber. Further, a stainless steel porous platen (McMaster-Carr, Douglasville, GA) with porosity = 0.95 comprised the bottom of the confined compression chamber, allowing fluid to flow out of and into the tissue during creep and recovery, respectively.

**Figure 5:**
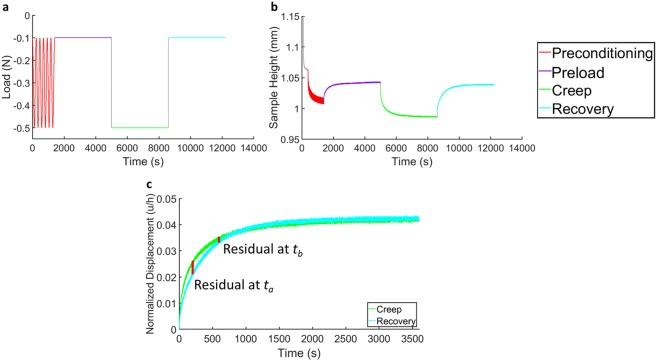
Confined compression test battery. (**a**) Applied load protocol. (**b**) Measured data from a representative explant. (**c**) Illustration of residuals from a representative explant for assessment of similarity of creep and recovery curves via calculation of the average residual across time.

Creep and recovery tests were performed in load control on an MTS Acumen 3 materials test system (MTS Systems Corporation, Eden Prairie, MN). The measured force and displacement data were recorded at a sampling rate of 50 Hz. The test battery included four steps: 1) cyclic preconditioning (peak-to-peak sinusoid of −0.1 N to −0.5 N at 0.10 Hz, 100 cycles), to ensure explants reached a repeatable steady-state level of hydration throughout the depth of the tissue^58^; 2) a preload (−0.1 N, 60 min), to allow explants to equilibrate to a baseline thickness under a small compressive load^39,47^; 3) a creep load (−0.5 N, 60 min), to measure the strain response during creep; 4) a recovery load (−0.1 N, 60 min), to measure the strain response during recovery (Fig. 5a). Notably, the preload and recovery levels (−0.1 N) were the same to ensure that the change in force occurring between steps 2 and 3 was equal in magnitude and opposite in direction to that occurring between steps 3 and 4. The preload, creep, and recovery loads were each applied at a rate of 0.05 N/s. Further, the magnitude of the change in force (0.4 N) during creep and recovery was chosen to result in less than 20% strain in the tissue at equilibrium^37,39,49^. Similarly, the preconditioning cycled between the same creep and recovery loads (−0.1 N and −0.5 N), and consisted of a 0.10 Hz sinusoid for 100 cycles as this rate and number have been shown to be sufficient for the tissue to reach a dynamic equilibrium in which no further ratcheting strain occurs per cycle^58^. Finally, the preload, creep load, and recovery load were each held for 60 minutes to allow the explants to reach equilibrium in each phase. When the recovery load was initiated, there was no evidence that the indenter pulled away from the explant. Specifically, negative force readings (indicating compression) were maintained throughout the testing.

### Data analysis

After testing, the data trace from each explant was checked to ensure that explants reached equilibrium. Equilibrium was defined as a change in explant height less than 0.6 microns over the final 60 seconds of creep, corresponding to a change in explant height less than 0.010 microns per second at the end of the creep phase^9,49^. All explants included in the analysis met this equilibrium criterion.

Next, the biphasic creep solution (Eq. 1)^37^ was fit to the measured creep and recovery deformation responses (Fig. 5b,c) separately to calculate the tissue’s characteristic time (*τ*_0_) and aggregate modulus (*H*_*A*_) during both the creep and recovery phases. The characteristic time (Eq. 1c) represents the time constant of the exponential term of Eq. 1a when n = 0, corresponding to the first term in the summation^55^. The fit procedure was performed in MATLAB (version R2018a, Mathworks, Natick, MA) using the nonlinear least-squares curve-fitting algorithm lsqcurvefit. Baseline thickness was defined as the mean explant thickness over the final five minutes of the preload phase (step 2 of the test battery, described above). Similarity between the creep and recovery response was assessed via the average residual, defined as the mean absolute error between the creep and recovery strain curves across time for a given explant, expressed as a percent of the final (60 minute) creep strain (Fig. 5c). Therefore, the average residual represents the expected error at any point in time between the creep and recovery curves for a given explant. Likewise, the mean average residual across multiple explants represents the mean expected error between the creep and recovery phases. Statistical analyses were performed in SAS (version 9.4, SAS Institute, Cary, NC) with p < 0.05 indicating significance. Outcome variables assessed were the aggregate modulus and characteristic time from each phase (creep and recovery). One-way repeated-measures analyses of variance (ANOVA) were performed to test for differences in outcome variables between the creep and recovery phases. Cartilage location (tibial plateau vs femoral trochlea) was included as a factor to examine differences in mechanical properties across location. Further, Pearson correlations were calculated between outcome variables. Data are summarized using the mean ± one standard deviation unless otherwise indicated.

1a $$\frac{u}{h}=-\,\frac{{\sigma }_{0}}{{H}_{A}}[1-2{\sum }_{n=0}^{\infty }\frac{1}{{\pi }^{2}{(n+\frac{1}{2})}^{2}}{e}^{-{\pi }^{2}{(n+\frac{1}{2})}^{2}\frac{t}{\tau }}]$$

1b $$\tau =\frac{{h}^{2}}{{H}_{A}k}$$

1c $${\tau }_{0}=\frac{4}{{\pi }^{2}}\frac{{h}^{2}}{{H}_{A}k}$$

where u = surface displacement, *h* = baseline thickness, *σ*_0_ = applied stress,

*H*_*A*_ = aggregate modulus, *τ* = time constant, *τ*_0_ = characteristic time, and

*k* = permeability.

## Data Availability

The datasets generated and analyzed during the current study are available from the corresponding author upon reasonable request.
